# Knowledge, attitudes, and preventive practices about colorectal cancer among adults in an area of Southern Italy

**DOI:** 10.1186/1471-2407-8-171

**Published:** 2008-06-11

**Authors:** Alessandra Sessa, Rossella Abbate, Gabriella Di Giuseppe, Paolo Marinelli, Italo F Angelillo

**Affiliations:** 1 Department of Public, Clinical and Preventive Medicine, Second University of Naples, Naples, Italy

## Abstract

**Background:**

Colorectal cancer (CRC) is the second most commonly diagnosed cancer for both sexes in developed countries. This study assessed the knowledge, attitudes, and preventive practices regarding CRC of adults in Italy.

**Methods:**

A random sample of 1165 adults received a self-administered questionnaire on socio-demographic characteristics; knowledge regarding definition, risk factors, and screening; attitudes regarding perceived risk of contracting CRC and utility of screening tests; health-related behaviors and health care use; source of information.

**Results:**

Only 18.5% knew the two main modifiable risk factors (low physical activity, high caloric intake from fat) and this knowledge was significantly associated with higher educational level, performing physical activity, modification of dietary habits and physical activity for fear of contracting CRC, and lower risk perception of contracting CRC. Half of respondents identified fecal occult blood testing (FOBT) as main test for CRC prevention and were more knowledgeable those unmarried, more educated, who knew the main risk factors of CRC, and have received advice by physician of performing FOBT. Personal opinion that screening is useful for CRC prevention was high with a mean score of 8.3 and it was predicted by respondents' lower education, beliefs that CRC can be prevented, higher personal perceived risk of contracting CRC, and information received by physician about CRC. An appropriate behavior of performing FOBT if eligible or not performing if not eligible was significantly higher in female, younger, more educated, in those who have been recommended by physician for undergo or not undergo FOBT, and who have not personal history of precancerous lesions and familial history of precancerous lesions or CRC.

**Conclusion:**

Linkages between health care and educational systems are needed to improve the levels of knowledge and to raise CRC screening adherence.

## Background

Malignant neoplasms from all cancers are the second leading cause of death after heart disease and colorectal cancer (CRC) is the third most commonly diagnosed form of cancer in each sex and the second for both sexes combined in essentially all economically developed countries [1]. At current rates, approximately 6% of individuals will develop CRC within their lifetime, and about half of them will die from this disease [2]. In the United States, it is estimated that 148,810 new cases of CRC will be diagnosed in the 2008 with 49,960 deaths [3], whereas in Italy in the period 2000–2003, there were 38,643 new cases [4] and, as regards mortality, in 2002 there were 29,734 deaths [5]. Due to the aging population and population growth, the expected numbers will increase in forthcoming years. Thus, prevention and early detection has immense public health importance. Indeed, the overwhelming evidence indicates that a vast majority of these cases and associated deaths could be reduced if diagnosed early enough and prevented by existing primary and secondary intervention. At least 70% of colon cancers may be preventable by focusing on modifiable risk factors and making moderate changes in diet and lifestyle [2] and secondary prevention is also critically important to prevent mortality. In the United States is recommended a fecal occult blood testing (FOBT) or lower endoscopy examination [3] and in Europe a FOBT [6]. In Italy, where a population based organized screening, in which all individuals in the target group are invited to take part, is under implementation, the majority of programs employ the FOBT [7,8].

A high participation level of the general population is essential for prevention programs to succeed. The key to achieving adequate compliance is informing the community through an educational campaign about the nature and extent of the disease, as well as preventive measures to be used. Relatively little is known regarding current knowledge [9], attitudes [9,10], and behaviors about CRC in the general population [10-19], and such understanding is imperative. Therefore, the goal of this study was to characterize the levels of knowledge, attitudes, and preventive practices about CRC among a sample of adults in an area of Southern Italy.

## Methods

A cross-sectional survey was conducted from November 2006 to March 2007. A random sample of 1165 parents of children attending three randomly selected public schools were recruited in the geographic area of the Campania region, in the South of Italy.

Before the study, permission and collaboration of the head of each school were obtained. A pilot study and pretest were carried out with a sample of 25 adults, similar to those included in the final study, in order to evaluate the comprehensibility of the wording of each question. Feedback was incorporated into the survey prior to the initial delivering. Data for the study were collected by self-administered anonymous structured questionnaire. The questionnaires were delivered, in sealed envelope, to the children in each classroom by trained research assistants and requested were made that the survey be completed by one parent only. Each family also received a letter containing information about the purpose and objectives of the study, that participation was strictly voluntary, that the data collected would not be used for anything except the research aim. Confidentiality of responses was assured, and an envelope to facilitate the return of the completed questionnaire was made available. A respondent's consent was taken into account while filling the questionnaire. The questionnaire was divided into five major parts for ease of administration [see Additional file 1]. The socio-demographic section focused on personal characteristics of respondents, such as age, sex, marital status, educational level, occupational position, whether they were living alone or not, weight, and height; self-rated health and their personal or familial history of CRC. Body Mass Index (BMI) was calculated from self-reported weight and height by dividing weight in kilograms by height in meters squared. The self-reported height and weight have been demonstrated to be reliable with test-retest analyses [20,21]. Self-rated health was assessed on a ten-point Likert-type scale, with responses ranging from 1 (poor) to 10 (excellent). Knowledge was explored by requesting parents to answer a number of questions including definition, risk factors, and screening tests of CRC. This section elicited responses in a variety of formats: open for the definition; "yes", "no", and "do not know" for the screening tests; and closed-end with categorical (yes or no) for the risk factors. In the attitude section, the participants were also asked whether or not him/her perceived themselves to be at risk for contracting CRC and their opinion about the utility of screening tests for its prevention. Beliefs were measured on a three-point Likert-type scale anchored by disagree and agree and statement about risk perception and utility of screening tests on a ten-point Likert-type scale, with responses ranging from 1 (not at all) to 10 (very much). Questions about health-related behaviors and health care use included whether or not respondents perform physical activities, have modified their dietary habits and/or physical activity for fear of contracting CRC, have received advice of performing FOBT, have participated in preventive activities about CRC, and have undergone a test. Finally, respondents were also asked about the source of information in close-ended questions with multiple answers possible.

The study was approved by the Ethics Committee.

### Statistical analysis

All statistical analyses were conducted in two steps. First, bivariate analyses tested the associations between potential explanatory variables and each outcome of interest by using appropriate test statistic. Second, variables associated with each outcome of interest with a *p*-value ≤ 0.25 in bivariate analyses were entered into four separate multivariable logistic and linear regression models, using a stepwise technique, for each of the main following outcomes of interest: knowledge of the two main modifiable risk factors of CRC (low physical activity, high caloric intake from fat) (Model 1); knowledge of FOBT as main test for CRC prevention (Model 2); positive attitude towards the utility of screening tests for CRC prevention (Model 3); appropriate behavior in undergoing FOBT if eligible or not undergoing FOBT if not eligible (Model 4). In all models, the independent variables included were the following: gender (male = 0, female = 1), age (continuous, in years), marital status (single/separated/divorced/widowed = 0, married = 1), number of other persons in the household (continuous), educational level (continuous, in years), occupational position (three categories: unemployed = 0, lower managerial, artisans, commercial = 1, high professional, managerial = 2), perception of personal health status (continuous), belief that CRC can be prevented (no = 0, yes = 1), participation in preventive activities about CRC (no = 0, yes = 1), physician as source of information about CRC (no = 0, yes = 1), and need of additional information about CRC (no = 0, yes = 1). The following variables were also included: modify the dietary habits for fear of contracting CRC (no = 0, yes = 1), performing physical activity (no = 0, yes = 1), and modify the physical activity for fear of contracting CRC (no = 0, yes = 1) in Model 1; knowledge of risk factors of CRC (low physical activity, high caloric intake from fat, polyps, familial history of precancerous lesions or of CRC) (no = 0, yes = 1), and advice received by a physician of performing FOBT (no = 0, yes = 1) in Model 2; knowledge of FOBT as main test for CRC prevention (no = 0, yes = 1) in Model 3; personal history of precancerous lesions and familial history of precancerous lesions or CRC (no = 0, yes = 1) and recommendation by a physician for undergone or not undergone FOBT (no = 0, yes = 1) in Model 4; BMI (continuous) and knowledge of the definition of CRC (no = 0, yes = 1) in Models 1 and 2; personal perceived risk of contracting CRC (continuous) and personal or familial history of precancerous lesions or CRC (no = 0, yes = 1) in Models 1–3; knowledge of the main unmodifiable risk factors of CRC (polyps, familial history of precancerous lesions or CRC) (no = 0, yes = 1) in Models 1 and 3; positive attitude towards the utility of tests for CRC prevention (continuous) in Models 2 and 4. The significance level for variables entering the logistic and linear regression models was set at 0.2 and for removing from the model at 0.4. Odds ratios (ORs) and 95% confidence intervals (CIs) were calculated with the use of logistic regression analyses. All of the tests for significance were two-sided and *p*-values ≤ 0.05 were considered statistically significant. All analyses were conducted using the Stata software program, version 8.1 [22].

## Results

A total of 595 subjects returned the self-administered questionnaire with an overall response rate of 51%. The principal characteristics of the study group are shown in Table 1. The mean age of participants was 44 years, two-thirds were females, one-third had a college degree, and 0.8% and 11.6% reported a personal or familial history of precancerous lesions or CRC, respectively.

**Table 1:** Socio-demographic characteristics and selected information about the study population

	*n*	*%*
*Gender*		
Female	396	66.6
Male	199	33.4
*Age (years)*	44.2 ± 5.8 (31–67)*	
*Marital status*		
Married	556	93.4
Other	39	6.6
*Number of other persons in the household*		
<3	64	10.8
3	341	57.3
4	138	23.2
≥5	52	8.7
*Educational level (years)*	14.2 ± 4*	
5–7	12	2
8–12	124	20.8
13–17	314	52.8
>17	145	24.4
*Occupational position*		
Unemployed	144	24.2
Lower managerial, artisans, commercial	346	58.2
High professional, managerial	105	17.6
*Perception of personal health status*	7.5 ± 1.6 (5–30)*	
*Personal history of precancerous lesions*		
No	590	99.2
Yes	5	0.8
*Personal history of colorectal cancer*		
No	594	99.8
Yes	1	0.2
*Familial history of precancerous lesions or colorectal cancer*		
No	526	88.4
Yes	69	11.6

*Mean ± Standard deviation (Range)

Table 2 summarizes the data concerning the level of knowledge about CRC risk factors and prevention strategies in the study population. An overall evaluation of the answers revealed a poor level of knowledge, with most of the respondents giving wrong answers. In particular, less than one-third (30.1%) were able to give the definition of CRC and a wide range of responses were given regarding the factors believed as potentially CRC causing. Thus, 24% to 62.9% correctly identified that low physical activity and polyps were risk factors for CRC and 54.1% to 99.3% that bowel infections and fruit and vegetable intake should not be risk factors. Overall, only 18.5% knew that low physical activity and high caloric intake from fat were the two main modifiable risk factors. The results of the knowledge about the preventive measures showed that 51.8% correctly identified FOBT as main test for CRC prevention. Results of the multiple logistic regression analysis indicated that five variables were statistically and independently associated with the knowledge that low physical activity and high caloric intake from fat were the two main modifiable risk factors: higher educational level (OR = 1.08; 95% CI 1.03–1.15), performing physical activity (OR = 1.79; 95% CI 1.14–2.83), modification of dietary habits (OR = 1.92; 95% CI 1.07–3.46) and of physical activity for fear of contracting CRC (OR = 2.22; 95% CI 1.1–4.49), and lower risk perception of contracting CRC (OR = 0.91; 95% CI 0.83–0.99) (Model 1 in Table 3). The second outcome of interest was the knowledge of FOBT as main test for CRC prevention and respondents were more likely to have this knowledge if they were unmarried (OR = 0.47; 95% CI 0.23–0.95), more educated (OR = 1.08; 95% CI 1.03–1.13), knew the risk factors of CRC (OR = 1.87; 95% CI 1.04–3.38), and had received advice by a physician of performing FOBT (OR = 3.43; 95% CI 2.01–5.83) (Model 2 in Table 3).

**Table 2:** Knowledge about colorectal cancer in the study population

	***Correctly answered***				***Do not know***	
	***Yes***	***%***	***No***	***%***	***n***	***%***
*Definition*	179	30.1	416	69.9	-	-
*Risk factors*						
Polyps	374	62.9	221	37.1	-	-
Familial history of colorectal cancer	321	53.9	274	46.1	-	-
High caloric intake from fat	279	46.9	316	53.1	-	-
Cigarette smoking	160	26.9	435	73.1	-	-
Low physical activity	143	24	452	76	-	-
*No risk factors*						
Fruit and vegetables intake	591	99.3	4	0.7	-	-
Hypertension	585	98.3	10	1.7	-	-
Oral contraceptives use	584	98.2	11	1.8	-	-
Diabetes	570	95.8	25	4.2	-	-
Bowel infections	322	54.1	273	45.9	-	-
*Preventive measures*						
Colonoscopy	373	62.7	106	17.8	116	19.5
Fecal occult blood testing	308	51.8	161	27	126	21.2
Double contrast barium enema	154	25.9	289	48.6	152	25.5
Sigmoidoscopy	62	10.4	360	60.5	173	29.1
*No preventive measures*						
Blood test	255	42.8	226	38	114	19.2
Ecography	249	41.9	222	37.3	124	20.8

**Table 3:** Multivariate logistic (1, 2, 4) and linear (3) regression analyses indicating associations between several variables and the different outcomes

Variable	OR	95% CI	*p*
**Model 1**. Knowledge of the two main modifiable risk factors of colorectal cancer (low physical activity, high caloric intake from fat)			
Log likelihood = -265.58, χ^2 ^= 38.51 (5 df), *p *< 0.0001			
Educational level			
Higher	1.08	1.03–1.15	0.004
Performing physical activity			
No	1.0*		
Yes	1.79	1.14–2.83	0.012
Physical activity modified for fear of contracting colorectal cancer			
No	1.0*		
Yes	2.22	1.1–4.49	0.027
Personal perceived risk of contracting colorectal cancer			
Lower	0.91	0.83–0.99	0.028
Dietary habits modified for fear of contracting colorectal cancer			
No	1.0*		
Yes	1.92	1.07–3.46	0.029

**Model 2**. Knowledge of fecal occult blood testing as main test for colorectal cancer prevention			
Log likelihood = -379.53, χ^2 ^= 65.05 (8 df), *p *< 0.0001			
Advice received by physician of performing fecal occult blood testing			
No	1.0*		
Yes	3.43	2.01–5.83	<0.001
Educational level			
Higher	1.08	1.03–1.13	0.001
Marital status			
Single/separated/divorced/widowed	1.0*		
Married	0.47	0.23–0.95	0.035
Knowledge of risk factors of colorectal cancer			
No	1.0*		
Yes	1.87	1.04–3.38	0.037
Participation in preventive activities about colorectal cancer			
No	1.0*		
Yes	3.19	0.83–12.27	0.09
Physician as source of information about colorectal cancer			
No	1.0*		
Yes	1.43	0.88–2.33	0.15
Belief that colorectal cancer can be prevented			
No	1.0*		
Yes	1.22	0.86–1.74	0.26
Personal or familial history of precancerous lesions or colorectal cancer			
No	1.0*		
Yes	1.36	0.8–2.31	0.26

**Model 4**. Appropriate behavior in undergoing fecal occult blood testing if eligible or not undergoing fecal occult blood testing if not eligible			
Log likelihood = -238.38, χ^2 ^= 219.35 (6 df), *p *< 0.0001			
Age			
Younger	0.85	0.81–0.9	<0.001
Personal history of precancerous lesions and familial history of precancerous lesions or colorectal cancer			
No	1.0*		
Yes	0.29	0.15–0.54	<0.001
Recommendation by a physician for undergone or not undergone fecal occult blood testing			
No	1.0*		
Yes	4.62	2.82–7.58	<0.001
Educational level			
Higher	1.07	1.01–1.13	0.034
Gender			
Male	1.0*		
Female	1.62	1.01–2.62	0.049
Number of other persons in the household			
Higher	1.14	0.87–1.49	0.34

Variable	Coeff.	t	*p*

**Model 3**. Positive attitude towards the utility of screening tests for colorectal cancer prevention			
F(6,588) = 12.95, *p *< 0.0001, R^2 ^= 11.7%, adjusted R^2 ^= 10.8%
Personal perceived risk of contracting colorectal cancer	0.11	3.65	<0.001
Belief that colorectal cancer can be prevented	1.13	6.87	<0.001
Educational level	-0.05	-2.73	0.007
Physician as source of information about colorectal cancer	0.49	2.27	0.024
Perception of personal health status	0.05	1.03	0.3
Gender	-0.16	-0.92	0.36
Constant	7.68		

*Reference category

With regard to the attitudes, 60.3% and 78.5% respectively responded that it is possible to prevent CRC and to treat the cancer in case of an early diagnosis. Participants were also asked to rate their perceived risk of contracting CRC and the mean score was 5.1, indicating low level of risk perception, with only 9.7% asserted a high degree of concern by answering "10". Personal opinion that screening is useful for CRC prevention was generally rated high with a mean score of 8.3 and 48.6% indicated a score of "10". Variables associated with the positive attitude towards the utility of screening for CRC prevention at *p*-value ≤ 0.25 at univariate level were entered into multivariable linear regression model. The most important variables that predicted this positive attitude were respondents' beliefs that CRC can be prevented and a high personal perceived risk of contracting CRC. Respondents with lower educational level and those who have received information about CRC from a physician were also significantly more likely to have this positive attitude (Model 3 in Table 3).

Of the survey respondents, 118 (19.9%) were eligible for performing FOBT according to the recommended Italian guidelines. With regard to participants' prior adherence to such recommendations, 72.7% reported having an appropriate behavior in undergoing FOBT if eligible or not undergoing FOBT if not eligible. Those younger (OR = 0.85; 95% CI 0.81–0.9), female (OR = 1.62; 95% CI 1.01–2.62), more educated (OR = 1.07; 95% CI 1.01–1.13), with no personal history of precancerous lesions and familial history of precancerous lesions or CRC (OR = 0.29; 95% CI 0.15–0.54), and who had been recommended by a physician to undergone or not undergone FOBT (OR = 4.62; 95% CI 2.82–7.58) were more likely to have had an appropriate behavior (Model 4 in Table 3). Of those who did not undergo the test, 19.5% were eligible, but the main reasons were lack of proper counsel by physicians and also that they felt healthy.

In terms of information, 60% of the respondents recalled receiving information regarding CRC and the most common sources had been the media (40.8%) and the physicians (15.8%). Interestingly, 75% of the respondents indicated that they would like more information about CRC.

## Discussion

This study sheds light on a group of adults in an area of Italy regarding the level of understanding community knowledge, attitudes, and preventive practices about CRC and it provides information for educators and policy makers that is necessary for guidance towards preventive campaigns. Previous research conducted in other countries exploring this topic typically focused on subjects aged fifty years or older [9-19]. Our study differs in that we analyzed a younger population and the main reason for our sample selection was that we felt that special attention should be paid to them because we are confident that prior behaviors in targeting promotion and information may have important public health implications in order to further increase understanding of CRC and performing appropriate preventive practices.

In the current study, the results indicated a general lack of knowledge and there are some important gaps. In regard to the responses to individual items, it is of some concern that fewer than one-third of the subjects surveyed were able to define CRC, the percentages of those correctly answering the questions on risk factors ranged from 24% for low physical activity to 62.9% for polyps, while 51.8% correctly identified FOBT as main test for the prevention of CRC. Previous studies conducted in other countries suggest a similar low level of knowledge about preventive measures. Indeed, in the United States, in a group of 104 patients ≥ 50 years of age from medical clinics, 69.2% and 49%, respectively, identified colonoscopy and FOBT as the main screening tests for the prevention of CRC [15] and in a population-based study of 105 white males 50–79 years of age, 75% had heard of colonoscopy [11]. Better awareness has been acknowledge in a group of 648 individuals aged 45–66 years in south-west England since 42.2% and 43.3% identified inactivity and smoking as risk factors, respectively [9]. Our findings that the respondents more educated, unmarried, with lower personal perceived risk of contracting CRC, and who had received advice by a physician were more likely to provide a correct response to the questions concerning modifiable risk factors and FOBT for CRC prevention suggest that information about CRC is not yet widely disseminated. The possible interpretation that those with lower personal perceived risk of contracting CRC were more likely to have a higher level of knowledge is that they are particularly motivated to acquire information at an early stage. Moreover, this is also supported by the association, although not statistically significant, with the participation in preventive activities and this emphasizes the crucial role of the physician in influencing patient knowledge.

Our findings concerning attitudes towards both the prevention of CRC in general and its screening tests in particular are encouraging, with a relatively high mean score on the utility of tests. Although respondents reported gaining information from a variety of sources, they again demonstrated very high levels of trust in physicians. Indeed, as revealed by multivariate analysis, this study demonstrates an association between information delivered by physicians and positive attitudes, since those identifying a physician as their primary source of information about CRC had the highest mean attitude score. So, because only 15.8% of respondents claimed to receive information about CRC from physicians, this avenue of support can be significantly strengthened. It is precisely this specific kind of information that might be required to satisfy the large percentage (75%) who indicated that they would need more information about CRC.

In our responders, the vast majority reported appropriate behavior regarding the periodicity with which subjects ought to be screened. Indeed, 72.7% reported having appropriate behavior in undergoing FOBT if eligible or not undergoing FOBT if not eligible according to Italian guidelines. The most frequently cited reasons by the respondents for not having yet undergone FOBT were that these had not been recommended by a physician and that they felt healthy. This finding is consistent with a study conducted in Canada among a group of relatives of CRC patients [13]. Still, it is troubling that 19.5% of those who never undergone FOBT were eligible. As expected, recommendation by a physician for undergoing or not undergoing FOBT has a positive influence to improve appropriate behavior.

The results of the study should be interpreted in light of some potential limitations. First, this was a cross-sectional study and as such it does not permit the establishment of a casual relationship between the different variables and CRC. Second, the use of self-administered questionnaires to measure behavior and perceptions, like all similar surveys, may allow the possibility either that the responses may be incomplete or may not reflect the truth with people who may have responded differently. We are confident that such problems are minimal because the questionnaires had not missing data and the responses were self-reported in a confidential and anonymous setting. Third, the representativeness of our sample may be limited by the response rate at 51%, though not uncommon in such studies, and it is equally possible that a non-response bias of those who responded may have characteristics that made them different from those who did not respond. Although the low response rate does not affect the internal validity of the findings, it may decrease the generalizability of the results. However, respondents did not differ significantly from the population of the same area with respect to the principal socio-demographic characteristics, thus the findings may be generalized.

## Conclusion

In summary, our data clearly indicate that linkages between health care and educational systems will be vital to improve levels of knowledge and understanding of CRC preventive measures and to improve screening adherence.

## Competing interests

The authors declare that they have no competing interests.

## Authors' contributions

AS participated in the design of the study, data collection, statistical analysis, and interpretation of the data. RA participated in the statistical analysis and interpretation of the data. GDG participated in the design of the study and data collection. PM participated in the design of the study and interpretation of the data. IFA, the principal investigator, designed the study, was responsible for the data collection, statistical analysis and interpretation of the data, and wrote the article. All Authors read and approved the final manuscript.

## Pre-publication history

The pre-publication history for this paper can be accessed here:



## Supplementary Material

Additional File 1 QuestionnaireColon: Questionnaire used in the survey. Click here for file
