# Supplementary material for: Knowledge, attitudes, and preventive practices about colorectal cancer among adults in an area of Southern Italy
Source: BMC Cancer. 2008 Jun 11;8:171. doi: 10.1186/1471-2407-8-171 (PMC2474634; doi:10.1186/1471-2407-8-171)
Supplement: Additional File 1 — QuestionnaireColon: Questionnaire used in the survey. [file 1471-2407-8-171-S1.doc]

## SOCIO-DEMOGRAPHIC CHARACTERISTICS

This section is designed to gather information about your socio-demographic characteristics.

**A1.** How old were you on your last birthday? _________

**A2.** What is your sex? □ Male □ Female

**A3.** What is your marital status? □ Married □ Single (never married) □ Other

**A4.** How many persons are there in your household? (not counting you) ___

**A5.** What is your highest education level? ______________________

**A6.** What is your occupation? ________________

**A7.** What is your weight? kg __________ **A8.** What is your height? cm __________

**A9.** How would you rate your current health status on a 1 to 10 scale with 1 meaning bad and 10 very good?

1 2 3 4 5 6 7 8 9 10

Bad Very good

**A10.** Has anyone in your family (counting you) been diagnosed to have bowel disease(s)?

□ No □ Yes (who _________________________ which disease(s) __________________________________)

## KNOWLEDGE

This section is designed to explore your knowledge related to colorectal cancer (CRC).

**B1.** How do you define CRC? _____________________________________________________________________________

**B2.** Which of the following is risk factor for CRC? (**mark one or more**)

**□** Iron deficiency **□** Bowel infections **□** High caloric intake from fat **□** Hypertension

**□** Oral contraceptives use **□** Low physical activity **□** Polyps **□** Cigarette smoking

**□** Radiation exposure **□** Diabetes **□** Familial history of CRC **□** Fruit and vegetables intake

**B3.** Which of the following is a screening test for CRC? (**if the response is yes, indicate how often should be performed and from what age**)

**Do not know No Yes**

- Blood test □ □ □ (how often________________ from what age _______)

- Double Contrast Barium Enema □ □ □ (how often________________ from what age _______)

- Abdominal ultrasound □ □ □ (how often________________ from what age _______)

- Sigmoidoscopy □ □ □ (how often________________ from what age _______)

- Fecal occult blood testing (FOBT) □ □ □ (how often________________ from what age _______)

- Colonoscopy □ □ □ (how often________________ from what age _______)

###### ATTITUDES

This section is designed to explore your attitudes towards CRC. Answer the following questions as truthfully as possible.

**C1.** How do you perceive your risk of contracting CRC on a 1 to 10 scale with 1 meaning no risk and 10 very much risk?

1 2 3 4 5 6 7 8 9 10

No risk Very much risk

**C2.** How would you rate the utility of screening tests for CRC prevention on a 1 to 10 scale with 1 meaning useless and 10 very useful?

1 2 3 4 5 6 7 8 9 10

Useless Very useful

**C3.** CRC may be prevented □ Agree □ Uncertain □ Disagree

**C4.** CRC may be treated when diagnosed at an early stage □ Agree □ Uncertain □ Disagree

###### BEHAVIORS

This section is designed to gather information about your behavior.

**D1.** In the last year have you modified your dietary habits for fear of contracting CRC?

□ No □ Yes (**please specify _____________________________________**)

**D2.** Do you perform any of the following physical activities?

|  | **No** | **Yes** |
| --- | --- | --- |
| Running |  |  |
| Swimming |  |  |
| Aerobic dance/Body building |  |  |
| Other (Please specify______________________) |  |  |

**D3.** In the last year have you modified your physical activity for fear of contracting CRC?

□ No □ Yes, I started □ Yes, I reduced □ Yes, I increased □ Yes, I stopped

**D4.** Who is the first physician you would consult for a health problem?

□ Primary □ Specialist □ Emergency hospital □ Hospital □ Other (**please specify _______________________**)

**D5.** Which exam has been recommended to you for CRC prevention? (**mark no, if not recommended**)

- Blood test □ No □ Yes (who recommended it? ____________)

- Double contrast barium enema □ No □ Yes (who recommended it? ____________)

- Abdominal ultrasound □ No □ Yes (who recommended it? ____________)

- Sigmoidoscopy □ No □ Yes (who recommended it? ____________)

- Fecal occult blood testing □ No □ Yes (who recommended it? ____________)

- Colonoscopy □ No □ Yes (who recommended it? ____________)

- Other exam (please specify) _____________________________ □ No □ Yes (who recommended it? ____________)

**D6.** Have you ever participated in preventive activities (information brochures, clinical tests, etc.) on CRC?

□ No □ Yes (which activity/ies ____________________________ organized by _____________________________)

**D7.** Which of the following exams for CRC prevention have you ever performed? (**answer to each exam**)

**D7.1 Blood test:** have you ever performed it?

□ **No** (indicate why) □ **I do not remember** □ **Yes**

□ It was not recommended Why: ____________

□ I have no health problems ________________

□ Long waiting lists How often: _______

□ Lack of time ________________

□ Fear of discovering a disease When last time: ____

□ I am not in the recommended age ________________

□ Fear of pain/embarrassment ________________

□ Other_______________________

**D7.2 Double contrast barium enema:** have you ever performed it?

□ **No** (indicate why) □ **I do not remember** □ **Yes**

□ It was not recommended Why: ____________

□ I have no health problems ________________

□ Long waiting lists How often: _______

□ Lack of time ________________

□ Fear of discovering a disease When last time: ____

□ I am not in the recommended age ________________

□ Fear of pain/embarrassment ________________

□ Other_______________________

**D7.3 Abdominal ultrasound:** have you ever performed it?

□ **No** (indicate why) □ **I do not remember** □ **Yes**

□ It was not recommended Why: ____________

□ I have no health problems ________________

□ Long waiting lists How often: _______

□ Lack of time ________________

□ Fear of discovering a disease When last time: ____

□ I am not in the recommended age ________________

□ Fear of pain/embarrassment ________________

□ Other_______________________

**D7.4 Fecal occult blood testing:** have you ever performed it?

□ **No** (indicate why) □ **I do not remember** □ **Yes**

□ It was not recommended Why: ____________

□ I have no health problems ________________

□ Long waiting lists How often: _______

□ Lack of time ________________

□ Fear of discovering a disease When last time: ____

□ I am not in the recommended age ________________

□ Fear of pain/embarrassment ________________

□ Other_______________________

**D7.5 Sigmoidoscopy:** have you ever performed it?

□ **No** (indicate why) □ **I do not remember** □ **Yes**

□ It was not recommended Why: ____________

□ I have no health problems ________________

□ Long waiting lists How often: _______

□ Lack of time ________________

□ Fear of discovering a disease When last time: ____

□ I am not in the recommended age ________________

□ Fear of pain/embarrassment ________________

□ Other_______________________

**D7.6 Colonoscopy:** have you ever performed it?

□ **No** (indicate why) □ **I do not remember** □ **Yes**

□ It was not recommended Why: ____________

□ I have no health problems ________________

□ Long waiting lists How often: _______

□ Lack of time ________________

□ Fear of discovering a disease When last time: ____

□ I am not in the recommended age ________________

□ Fear of pain/embarrassment ________________

□ Other_______________________

**E. INFORMATION**

**E1.** From which of the following sources do you receive information about CRC? (**mark one or more**)

□ None □ Scientific journals □ Mass-media □ Educational courses □ Physicians □ Other (**please specify ___________**)

**E2.** Do you feel you need more information about CRC? □ Yes □ No
